# A bioinformatics knowledge discovery in text application for grid computing

**DOI:** 10.1186/1471-2105-10-S6-S23

**Published:** 2009-06-16

**Authors:** Marcello Castellano, Giuseppe Mastronardi, Roberto Bellotti, Gianfranco Tarricone

**Affiliations:** 1 DEE Dipartimento di Elettrotecnica ed Elettronica, Politecnico di Bari, via Orabona, 4, 70125, Bari, Italy; 2 Istituto Nazionale di Fisica Nucleare Sezione di Bari e Dipartimento Interateneo di Fisica "M. Merlin", via Orabona, 4, 70125, Bari, Italy

## Abstract

**Background:**

A fundamental activity in biomedical research is Knowledge Discovery which has the ability to search through large amounts of biomedical information such as documents and data. High performance computational infrastructures, such as Grid technologies, are emerging as a possible infrastructure to tackle the intensive use of Information and Communication resources in life science. The goal of this work was to develop a software middleware solution in order to exploit the many knowledge discovery applications on scalable and distributed computing systems to achieve intensive use of ICT resources.

**Methods:**

The development of a grid application for Knowledge Discovery in Text using a middleware solution based methodology is presented. The system must be able to: perform a user application model, process the jobs with the aim of creating many parallel jobs to distribute on the computational nodes. Finally, the system must be aware of the computational resources available, their status and must be able to monitor the execution of parallel jobs. These operative requirements lead to design a middleware to be specialized using user application modules. It included a graphical user interface in order to access to a node search system, a load balancing system and a transfer optimizer to reduce communication costs.

**Results:**

A middleware solution prototype and the performance evaluation of it in terms of the speed-up factor is shown. It was written in JAVA on Globus Toolkit 4 to build the grid infrastructure based on GNU/Linux computer grid nodes. A test was carried out and the results are shown for the named entity recognition search of symptoms and pathologies. The search was applied to a collection of 5,000 scientific documents taken from PubMed.

**Conclusion:**

In this paper we discuss the development of a grid application based on a middleware solution. It has been tested on a knowledge discovery in text process to extract new and useful information about symptoms and pathologies from a large collection of unstructured scientific documents. As an example a computation of Knowledge Discovery in Database was applied on the output produced by the KDT user module to extract new knowledge about symptom and pathology bio-entities.

## Background

The progress in biomedical field largely relies on results which are obtained both in laboratories and institutions from around the world and published in several journals. With the amount of publications increasing daily, the problem of searching for highly specific data is getting more difficult. As one of frequent activities for the study of biomedicine, bio-entity recognition is receiving greater attention. Bio-entity recognition aims to identify and classify technical terms corresponding to the instances of concepts that are of interest to molecular biologists. Examples of such entities include the names of proteins, genes, their locations of activity such as the names of cells or organisms, drugs, symptoms, pathologies and so on. Entity recognition is becoming increasingly important with the massive increase in reported results due to high throughput experimental methods. It can be used in several higher level information access tasks such as relation extraction, summarization and question answering. Recognising biological entities in texts allow further extraction of relationships and key concepts of interest and allowing those concepts to be represented in some consistent, normalised form. This task is challenging for several reasons, because a complete dictionary of biological entities does not exist, hence, simple text matching algorithms do not produce reliable results. In addition, the same word or phrase can refer to a different thing depending upon the context and some biological entities have several names. Moreover, biological entities can have multi-word names which can complicate the task with the need to determine name boundaries and resolve the overlap of candidate names. Because of the potential utility of this recognition and the complexity of the problem, named entity recognition has attracted the interest of many researchers, and generated much research. With the large amount of genomic information being generated by biomedical researchers, it should not be surprising that in the genomics era, much of the work in biomedical name-entity recognition has focused on identifying gene and protein names in free text [1,2].

Although the search problem has been simplified by search engines, the number of results returned is usually very large, while the relevance of the results may be small. The search based on keywords is unable to answer specific questions about the location and usage of the keywords in retrieval documents. For all these reasons the problem of discovering useful knowledge from unstructured text, is attracting increasing attention. The solution of this problem is called Knowledge Discovery in Text and it refers to the process of extracting interesting and not-retrieval patterns or knowledge from unstructured text documents. The application of Knowledge Discovery in Text in the biomedical field can improve efficiency for researchers by shifting the burden of information overload from them to the computer by applying Text Mining (TM) automatic procedure. TM examines the relationships between specific kinds of information contained in a single document or across a whole volume of documents. For example, TM can aid database curators by selecting the articles most likely to contain information of interest. This could then lead to the discovery of potential treatments for migraines by looking for pharmacological substances that are associated with biological processes about migraines. Knowledge discovery in text and applications of this process are available in the literature [3-6].

The problems of application based on the mining methods, described so far, often occur in data-intensive situations. These situation require that the same logic be applied to a large collection of different data independent from each other. Hence, the limits will be technological if these problems are addressed by traditional machines that sequentially perform the same set of instructions on an entire collection of homogeneous and independent data. The time required for execution will increase according to the size of the collection, hence, this will become the limiting factor in these applications. For awhile now, computing literature was offered possible solutions by proposing for parallel calculating like SIMD. This latter supercalculator, however, regards expensive centralized computing systems. A more economic solution with dynamic scaling characteristics according to the size of the data collection to be analyzed, is offered by systems weakly linked to calculating networks. Recently, a computational paradigm is being explored which suggests creating computer technology pools. These pools have a high use efficiency and can achieve performance levels comparable monolithic calculating systems, i.e. supercomputers. The use of this technology is called Grid Computing. The type of computing is based on the use of a basic middleware infrastructure on which a middleware solution is constructed. In other words, services which orient the infrastructure to a specific class of use. Much effort is being made in Europe and internationally to develop this calculating tool for users in the fields of physics, biology and research in general [7-9].

Bio-medical informatics is one of the areas in which Grid technology advances could bring significant benefit for the search studies of scientists well as the everyday work of clinicians. Recently, there has been much excitement in the distributed and parallel systems community as well as that of distributed database applications in the emergence of Grids as the platform for scientific and medical collaborative computing. Grid computing promises to resolve many of the difficulties in facilitating medical informatics and medical image analysis by allowing radiologists and clinicians to collaborate without having to co-locate. Grid technology can potentially provide medical applications with an architecture for easy and transparent access to distributed heterogeneous resources, like data storage, networks, computational resources, across different organizations and administrative domains. The Grid offers a configurable environment whereby structures can be reorganized dynamically without affecting any overall active Grid processing. In particular, the Grid can address the following issue relevant to bio-medical domains: data distribution, that is, the Grid provides connectivity for medical data distributed over different sites heterogeneity, that is, the Grid addresses the issue of heterogeneity by developing common interfaces for access and integration of diverse data sources; data processing and analysis, that is, the Grid offers a platform for transparent resource management in medical analyses; security and confidentiality, that is, enabling secure data exchange between hospitals distributed across networks which is one of the major concerns of medical applications [10-13]. Even though the projects at international European and National levels attempt to achieve these goals on a large scale, work which reconstructs the scenario on a small scale can allow laboratory analyses through the testing of small problems which occur like the experimentation of new analytic procedures at the application level.

In this work, we present a feasibility study to build a middleware for SIMD applications. Their performance is demonstrated with a case study of named bio-entity recognition. The application is based on the knowledge discovery in text to annotate new knowledge from unstructured textual documents. Moreover, the middleware offers the ability to perform the application in a distributed environment using grid computing. In particular, software platform GATE was used to perform automatic analysis of scientific documents. GATE is a toolkit used with the GATE Java API and its documentation is available in [14,15]. Globus is a toolkit which enables the construction of middleware grid services oriented towards data-intensive applications. A large amount of documentation is available in [16]. Finally, it should be noted that new knowledge discovery procedures could applied to the results of textual analyses to generate new knowledge. An example of this is shown with an application known Knowledge Discovery in Database (KDD). The study for the development of a middleware solution which little by little can supply the user with more and more instruments for the analysis of knowledge discovery could define new knowledge discovery procedures. These developments would be of great use for studies in fields such as bio-medicine.

## Methods

The bioinformatics application, discussed in this paper, concerns the extraction of biological entities related to symptoms and pathologies from a large collection of biomedical papers. In addition, the application searches for new knowledge about them using the knowledge discovery in text for grid computing. In this section, we briefly describe the KDT methodology and then we explain how to simplify a data-intensive application in a SIMD scalable job from the data and cpu computational resource point of view in a grid environment.

**Figure 1:**
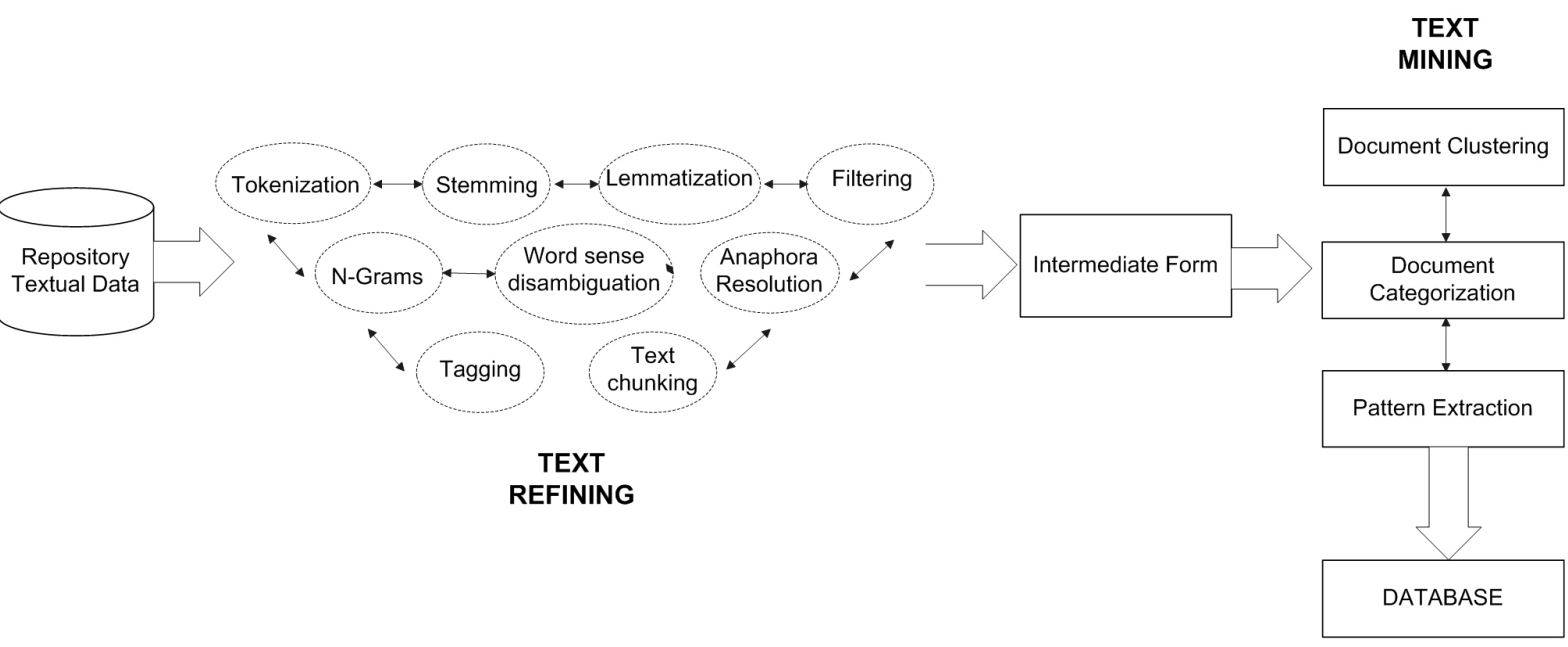
**Knowledge discovery in text process**. This figure shows the Knowledge Discovery in Text process. It is composed by *Text Refining *and *Text Mining *phases. The former transforms a free-form text document into a chosen Intermediate Form while that latter deduces patterns or knowledge from the Intermediate Form. *Text Refining *input are not-structured data such as texts or semi-structured data like HTML pages. It consists of *Tokenization*, which splits a text document into a stream of words by removing all punctuation marks and by replacing tabs and other non-text characters with single white spaces, and *Filtering *methods, which remove words like articles, conjunctions, prepositions, etc. from the documents. *Lemmatization *methods try to map verb forms to the infinite tense and nouns to their singular form. *Stemming *methods attempt to build the basic forms of words, for example, by stripping the plural 's' from nouns, the 'ing' from verbs, or other affixes. Additional linguistic pre-processing may be needed to enhance the available information about terms: *N-grams individualization*, which is n-word generic sequences that do not necessarily correspond to an idiomatic use; *Anaphora resolution*, which can identify relationships among a linguistic expression (anaphora) and its preceding phrase, thus, determining the corresponding reference; *Part-of-speech tagging *(POS) determines the part of speech tag, noun, verb, adjective, etc. for each term; *Text chunking *aims at grouping adjacent words in a sentence; *Word Sense Disambiguation *(WSD) tries to resolve the ambiguity in the meaning of single words or phrases; *Parsing *produces a full parse tree of a sentence (subject, object, etc.). *Text Refining *output can be stored in database, XML file or other structured forms which are referred to as the Intermediate Form. *Text Mining *techniques are then applied to the Intermediate Form. The Text Mining phases are: *document clustering*, *document categorization*, and *pattern extraction*.

Knowledge Discovery in Text refers generally to the process of extracting interesting information from a large amount of unstructured texts. Specific methods and algorithms are required in order to extract useful patterns. Text mining is the application of these algorithms and methods from the fields of machine learning and statistics to texts. The goal is to find useful patterns. To mine large document collections it is necessary to pre-process the text documents and store the information in a data structure, which is more appropriate for further processing than a plain text file. Although, several methods try to also exploit the syntactic structure and semantics of text, most text mining approaches are based on the idea that a text document can be represented by a set of words. That is to say, a text document is described based on the set of words contained in it. Figure 1 shows the KDT process based on two phases: *Text Refining *or *Text Pre-processing *and *Text Mining*. The central element of the Text Mining process is understanding the concepts being presented in the text. The process not only considers the presence or frequency of a word in a document but also aims at finding the relationship between them. By doing so, the mining process attempts to find information contained within a text. The Text Mining phases are: *document clustering*, *document categorization*, and *pattern extraction*. *Document clustering *is the assignment of a multivariate entity to a few categories (classes, groups) previously undefined. The goal is to gather together similar entities. Textual clustering is used as an automatic process which divides a collection of documents into groups. Inside these groups, the documents have similarities based on selected characteristics: author, length, dates, keywords. Textual clustering can be used to provide a planning of the contents of document collections, to identify hidden similarities, to facilitate the process of browsing and to find correlated or similar information. If the clustering works with keywords or features that represent the semantics of the documents, the identified groups will be distinguished on the basis of the different topics being discussed in the corpus. In *Document categorization*, the objects must be attributed to one or more classes or categories which will have already been identified. Classification is the process in which meaningful correlations among frequent data are identified. There are association rules for Text Categorization. All algorithms operate in two phases to produce association rules. First, all the whole keywords with greater or equal support with respect to the reference are listed to create what is called the frequent set. Then, all the association rules, that can be derived from the frequent set and that satisfy the given confidence, are established. In *Pattern Extraction*, some patterns are identified following the analysis of associations and tendencies. The discovery of associations is the process in which meaningful correlations among frequent whole data are found. Predictive Text Mining is used for identifying the tendencies in collected documents during a given time period while Association Analysis identifies the relationships among the attributes, for example, if the presence of a pattern implies the presence of another pattern in documents. [17-21]

**Figure 2:**
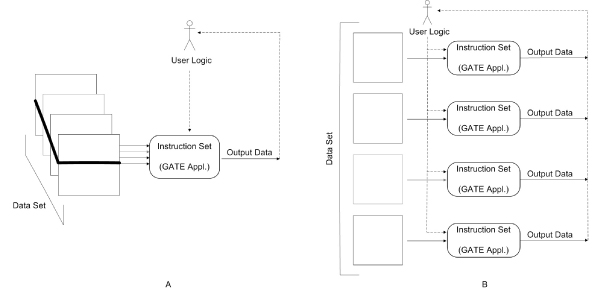
**Text mining job models (A – B)**. Figure 2A shows a schema of a typical Job produced by GATE. The tool consists of a framework and graphical interface that drives the user in the Text Mining operations. For example, the user defines a corpora instance which consists of a large number of text documents. Then GATE processes it according to the user's commands for carrying out a suitable text mining process. This is a single-instruction and multiple-data stream Job that reveals an intensive use of the CPU resource. Figure 2B shows the Job model which was adopted to overcome the single CPU bound. It distributes several computing nodes commanded by the user and a slice of the whole data set. At completion, each node produced results.

**Figure 3:**
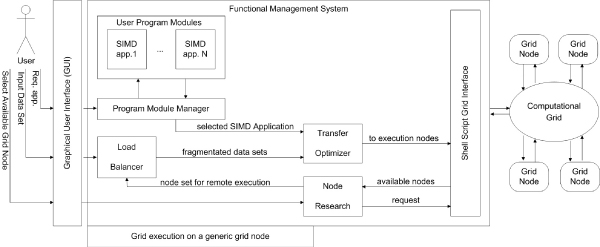
**Grid Middleware functioning**. This figure shows how the different parts of the system interact among each other to complete the objectives of the Grid Middleware. To start the system, the end user interacts with the node search system through the interface. Using calls through shell script to Condor scheduler, the node search system retrieves information on nodes in a computational grid and submits them to the user. The user selects the nodes for computation and specifies the input data set and the local directory that will contain the remote computing results. At this point, the user selects the SIMD application that will be distributed in the grid from a lists of SIMD Applications. Once these specifications have been fixed, the load balancer analyzes the input data set and the node set, which are to be used for the remote computing, and provides a peer distribution of the workload on the different nodes of the grid computing. After carrying out this subdivision, the transfer optimizer makes a compression of the data set and instruction set to send to different remote nodes. Following the compression operations, the remote computation on the different grid system nodes is started by calls to the shell script system. At the end of the remote computing, each node performs local compression of the computing results, and at this point, middleware recovers the compressed results and makes them available in decompressed form on the end user node.

The middleware solution allows the user to move from the situation in Figure 2A situation to that in Figure 2B. To achieve these goals the system will operate on an open source grid infrastructural middleware based on a data-intensive grid toolkit solution. The operations required of the system are: locating, submitting, monitoring and deleting remote jobs on Grid-based computer resources; a reliable data transfer which is optimized for high-bandwidth wide-area networks; a transfer optimization; a node discovery; a load balancing; the management of the User SIMD Applications and finally a simple Graphical User Interface (GUI). Figure 3 shows the functional components of the system and the organization of them. The computational grid with its services is the physical layer. It interacts with the system with the shell scripts. The functional management system layer, obtains the user requests with the graphical user interface and executes them using the shell script level parameterization and invocation. GUI allows the user to specify: the applicative module that will be distributed on the Grid, the data set on which the user module will be executed and the computational grid nodes. The user selects the SIMD application in order to perform the job by specifying the data set and the directory where the result produced by the grid can be stored. Data and the instruction set are sent to the remote nodes after being compressed by the transfer optimizer. Next, remote computation on the several grid system nodes begins. At the end of the remote computing, each node locally compresses the computing results, and the system makes the results available to the user. The functional management system is composed by several components that interoperate. The node research system is based on a scheduler to obtain all the information about the grid nodes' status, which allows a dynamic management of the grid nodes. The Load Balancer maintains a reasonable workload sharing between the grid nodes selected by the end user for the grid computation. The user job is processed analyzing the input data set and the computing node set with the aim to transform a single job in parallel jobs. The system propose a *SIMD applications management component *to extend the system applications based on the plug-in approach. After a preliminary phase in which the new application is linked to the system, the end user can select it with the GUI. It is well known that a critical factor for the elapsed-time of grid distributed applications is the time required by communications. For our applications, the communications consist of network data transfer (input data set) and instructions (instruction set) and then the remote computing results download in the user node. In order to optimize the communication time, the data is compressed to significantly reduce the data dimension run on the network. The compression factor and the advantage of this technology depend on the source data type. The transfer optimizer produces compressed packages containing a data set and an instruction set. Before being sent to remote nodes, the compression is also applied when the results computation return. The Shell Script Grid Interface consists of a collection of script modules used to start a remote or local job and to allow the parallel execution of calls [22-24].

## Results

**Figure 4:**
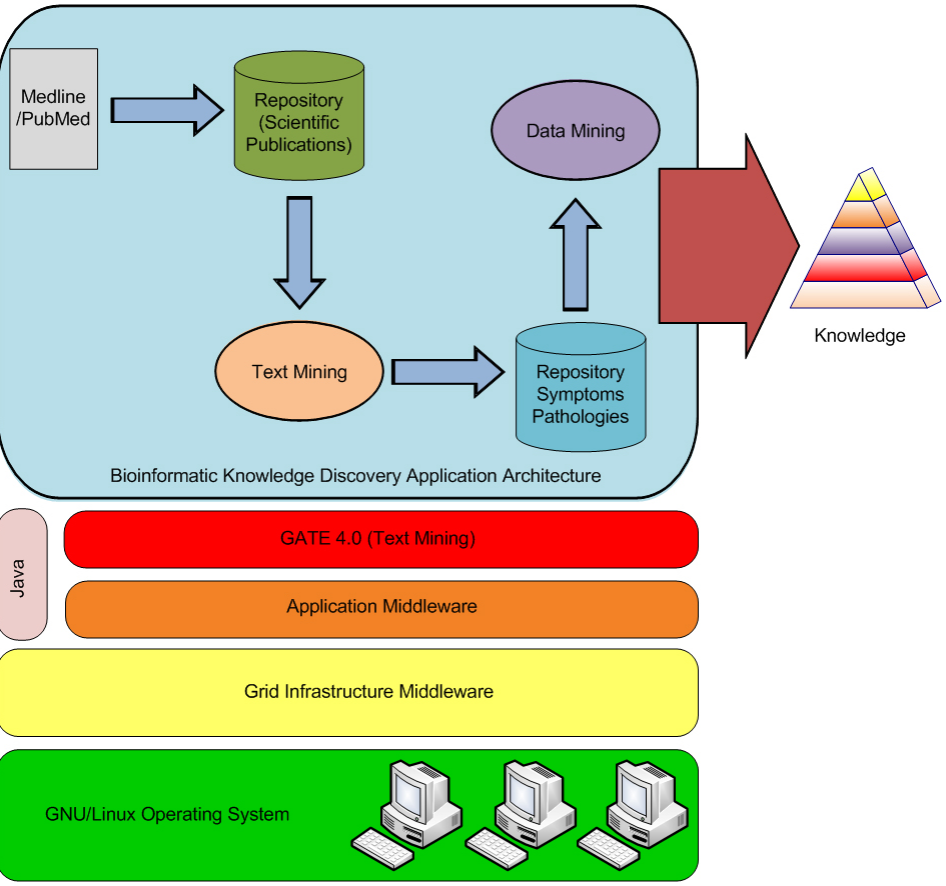
**Bioinformatics architecture**. This figure shows a bioinformatics knowledge discovery application architecture. It presents the integrated development environment, GATE, which was used for the text mining process. GATE operated on a collection of scientific publications The process of Text Mining starts from a set of scientific publications in full text available on MedLine/Pubmed (in pdf format). Moreover, the figure shows the Layer Architecture consisting of GATE 4.0 Toolkit for Text Mining, our Middleware solution written by Java API, the grid infrastructure middleware, and, finally, a physical layer that consists of a Gnu/Linux Operating System.

**Figure 5:**
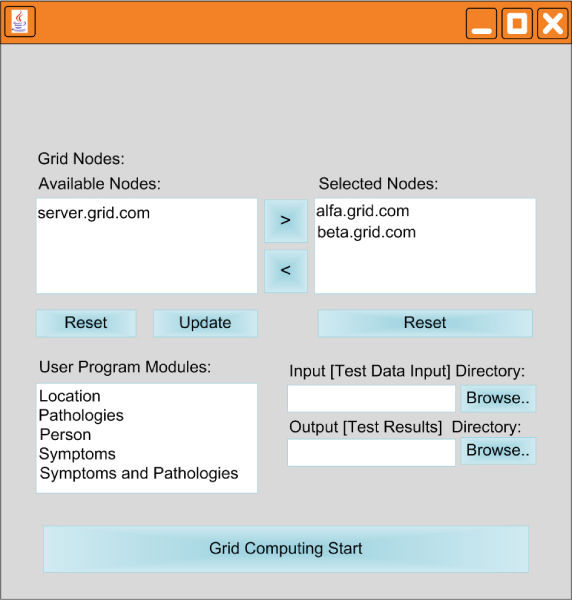
**Graphical user interface**. This figure shows the graphical user interface of the prototype. On the upper side, there is the user dialogue section with the node search system, the "update" press loads in the "available nodes" list all the nodes identified by search module as available on the grid. The special keys, on the right of the list, allows the user to choose the nodes which the user prefers proper for the computation. They will then appear in the "selected nodes" list. In the user program module the available applications, which must be executed on the selected nodes, appear. The user specifies the input/output directory for the job using the browse buttons.

The results of the feasibility study concern both the technological choices adopted for the construction of the prototype and the demonstration of these through the use of bioinformatics applications. The latter is in relation to the study of the knowledge discovery of bio-entities which included symptoms and pathologies contained in a collection of 5,000 documents. Figure 4 shows the system prototype's architecture with reference to the technological choices adopted. In particular, the software platform GATE was utilized for the knowledge discovery in text. The infrastructure of the resources of the calculations based on the computational grid was created with Globus toolkit. Finally, the middleware solution system referred to a code developed in Java Language through the Java Virtual Machine. Moreover, it expressed the actual grid requests with Linux Shell Script components and implements its internal services related to the job management functions through calls to the services available from GRAM, GridFTP and Condor System. GRAM enables the remote execution management where there is reliable operation, statefull monitoring, credential management and file staging. GridFTP provides high-performance, secure technologies for reliable data transfer, while the Condor System is a specialized workload management system for compute-intensive jobs. It provides a job queueing mechanism and a scheduling policy. Moreover, the middleware solution is able to manage different User SIMD Application Modules. These modules give a codified description of the program, which is to be performed, through operational requests of knowledge discovery made available by the environment that is being used. The UAMs are built with the use of a template. Figure 5 shows the GUI of a prototype accessable to a user for communicating the job to the system

**Figure 6:**
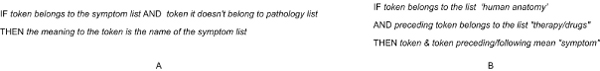
**Symptom rule pseudo code (A – B)**. This figure shows Symptom rule pseudo code. This rule is based on symptom keyword lists that contain only names of symptoms, The execution rule consists of matching the tokenized Document with largely used reference rule lists. The matching rule is what seeks the presence of each considered token and associates to that token in a keyword lists, and gives the name of the token to the same list, as shown in Figure 6A. The problem is when the token is present in more or no lists, in such cases grammatical rules and interpretation rules are necessary. An example of rule of such a case is shown in Figure 6B.

**Figure 7:**
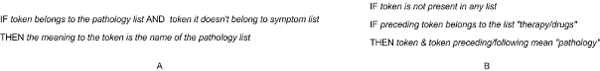
**Pathology rule pseudo code (A – B)**. This figure shows Pathology rule pseudo code. This rule is based on pathology keyword lists that contain only names of pathologies. The execution rule consists of matching the tokenized Document with largely used reference rule lists. The matching rule is what seeks the presence of each considered token and associates to that token in a keyword lists, and gives the name of the token to same list, as shown in Figure 7A. The problem is when the token is present in more or no lists, in such cases grammatical rules and interpretation rules are necessary. An example of rule of such a case is shown in Figure 7B.

The experimental set-up used to execute the test-run of the prototype was as follows.:

*a. Using the GATE plug-in StandAloneAnnie.java template file *[15,25]*the bioinformatics application for pathology and disease recognition was written as a java module and integrated into the system;*

b. A data collection consisting of 5,000 medical publications in text files format was created by PubMed Central Repository

c. A list of keyword about Symptoms and another keyword list about Pathologies have been created by  and  respectively. They were specified as LST file as required by the template file of ANNIE Plug-in for GATE;

*d. The rules on which GATE worked were defined in terms of exact matching with keyword lists and described using the JAPE template file of ANNIE Plug-in for GATE. Figure *6*and Figure *7*show an example of pseudo-code rule specification*.

e. A computational grid was based on three computational nodes, the Server, Alfa and Beta, Gnu/Linux machines operative on 100 Mbps Ethernet LAN were created using Globus Toolkit 4.0.5 with the access interface for Condor on the pool. Prior to this, the Condor scheduling system had been installed for each machine. The follows grid services were configured: GridFTP, for file transfer, GRAM for resource management and job submission, the MDS monitoring and discovery system (the information services component of Globus) and RTF for the secure file transfer operating solely on the server node, which is a central grid node for the reliable transfer management

*f. GATE installation and configuration on all grid nodes*.

**Figure 8:**
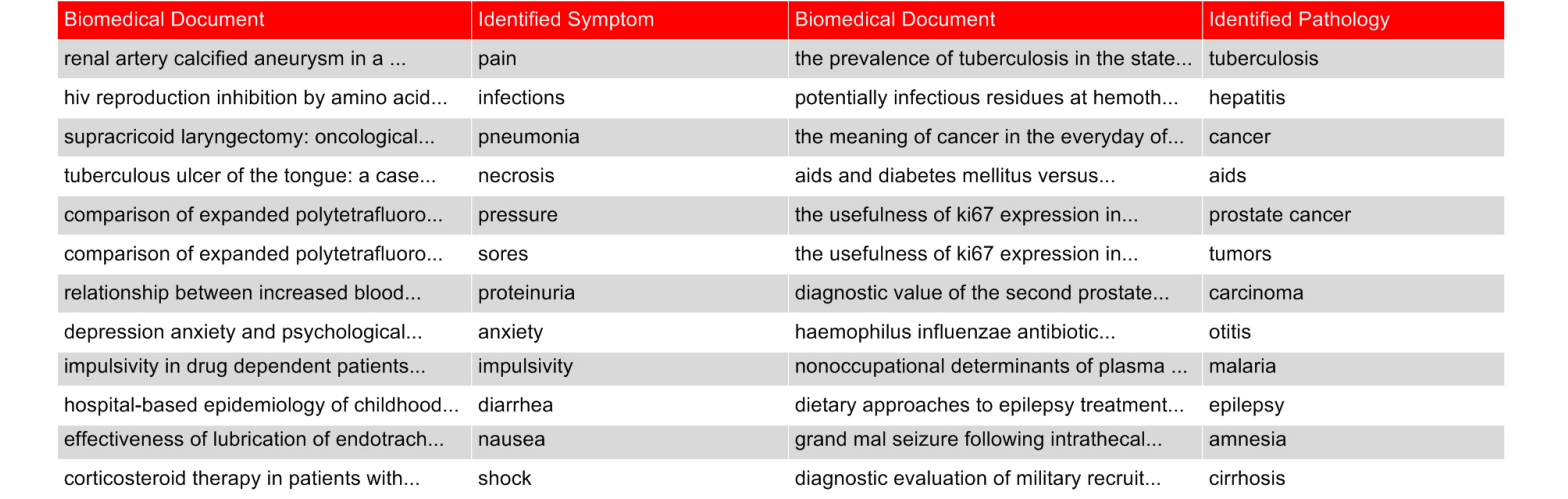
**Symptoms and pathologies**. This figure presents a part of the results of a knowledge discovery in text process application.

**Figure 9:**
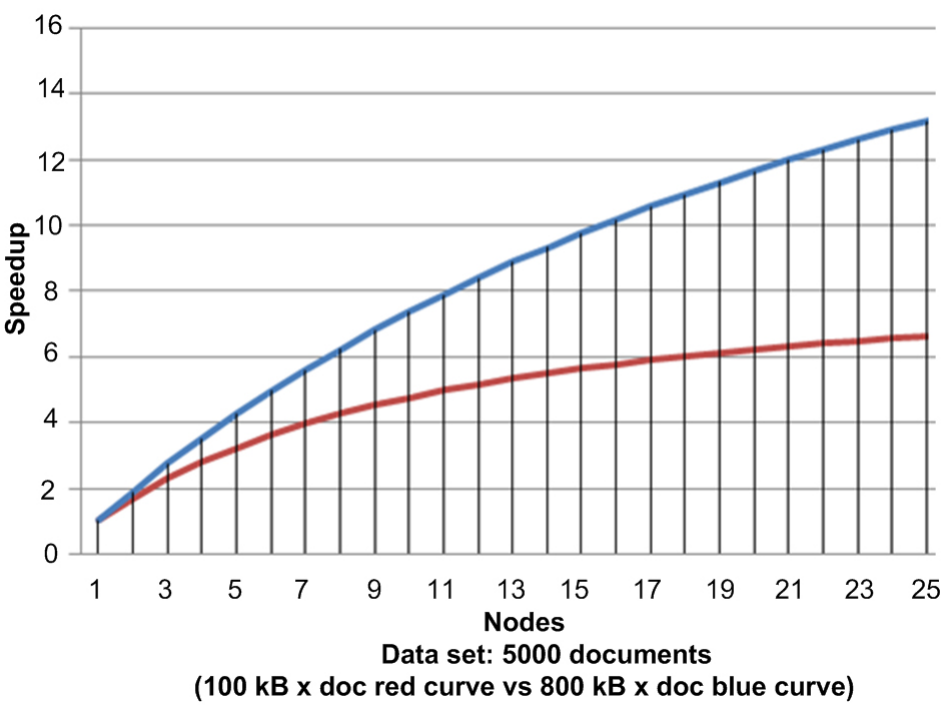
**Speedup coefficient versus node multiplicity**. This figure illustrates the trend of the speedup coefficient in correspondence to the growth of the node multiplicity. In the first case, a data set consisting of 5,000 documents was used, each having a size of 100 KB (red curve). In the second case, the data set consisted of 5,000 documents, each one with a size of 800 KB (blue curve).

*The test run started with a user logging *onto a generic grid node and launching the system which showed the Graphical User Interface. Then the user specified the correct program module to be performed as well as the data set collection and the output directory. Next, the user interacted with the node search system which, after query, showed a node list in the "Available Nodes" window for the computation. The user selected the nodes needed to execute the required user module and then used the "update" button to update the "Selected Nodes" window. Figure 5 shows what appears on the graphical user interface of the prototype after the user interacts to describe the test-run. The system was then ready to start the SIMD computation on the grid and the *Text mining process was performed on the biomedical papers according to the job description specified in the user module. *A part of the text mining results are show in Figure 8 as the real output produced by the system. We evaluated the contribution in terms of the time needed by the grid for the application. First, a run-time module of the input data set, which consisted of 5,000 documents, was evaluated on a traditional serial machine as follows:



Then, the data upload and data download times and user program module on the grid nodes were calculated by performing a simple mathematical calculation. Considering the typical ADSL bandwidth and the user program module run-time on the one node, the total run-time on the grid was:



Let:



The speedup factor referred to how much faster a parallel algorithm was in comparison to a corresponding sequential algorithm using the grid. The factor was computed for a node number ranging from 1 to 30 for various size documents. Figure 9 shows the trend of the speedup coefficient in correspondence to the growth of the node multiplicity. It shows that the use of the proposed middleware gave a real advantage when applied to jobs treated as SIMD that find real implementation on distributed systems like those proposed by a computational grid. This benefit was more noticeable with the increased size of a document.

## Discussion

This study began from the standpoint that, in biological research, new finding can be expressed through the analysis correlated, unstructured information present in publications and scientific documents. The application executed in this study adapted the Knowledge Discovery in Text process to the task of extracting biomedical knowledge, in terms of symptoms and pathologies. This facility could be a profitable support for physicians and medical researches needing to make important decisions. The strong points of the proposed system are that it can be used for applications in which the data can be partitioned into different and independent data-sets. Moreover, another fundamental characteristic of the proposed system was the grid-based approach, which was to be able to supply high performance computing infrastructures to satisfy computational problems in this field. Finally, we believe it is useful to emphasize that the knowledge discovery process in text should be considered one phase in a larger knowledge discovery program. Here, we have briefly reported a part of the finding obtained by applying to the knowledge output from KDT a further important process of Knowledge Discovery in Database (KDD). The field of KDD includes a new generation of techniques and tools for the automatic and intelligent analysis of large volumes of data, "data mines", in order to extract hidden knowledge.

**Figure 10:**
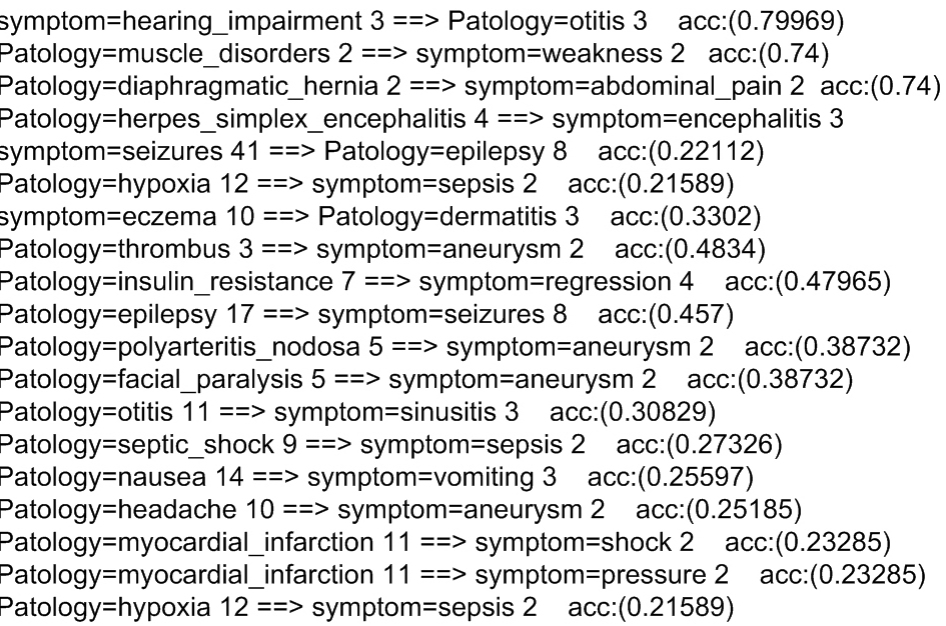
**Associative rules**. This figure illustrates the presentation seen by the user of the Knowledge Discovery in Database process output, i.e. the visualization of the process results. These results are obtained by applying Predictive Apriori algorithm. Its goal is to discover probabilistic associative rules between the data base records. Particularly, it tries to increase as much as possible, the correct associative relationship through a binomial distribution, in which the analyzed attribute is classified as correct or wrong. The results are associative rules with a probability percentage.

KDD is a process of identification of patterns and charactering trends on data. The trends have a certain level of general validity which are not taken for granted or noted but are potentially useful and easily understood. Numerous studies discuss the application of these techniques to biomedical studies. The findings presented here were obtained using the Waikato Environment for Knowledge Analysis or WEKA [26,27]. They show a further depth of knowledge extractable through the use of symptoms and pathologies, as reported in Figure 10. The final consideration could benefit from the development of a middleware solution specializing in more generalized cases of knowledge discovery. To achieve this objective, the next prototype will involve the integration of User Application Modules oriented for KDD based on the WEKA4WS [28]. The latter is designed to take advantage of a computational grid environment to propose data mining analyses, which are different on the same groups of data, for the creation of optimized models.

## Conclusion

In this paper we have presented the development of a middleware solution for a Bioinformatics Knowledge Discovery in Text process. It was designed for medical text documentation using a testbed computational Grid based on Globus middleware. We have discussed a Knowledge Discovery in Text process performed on medical papers with the purpose of identifying all the specific names for biological entities with particular attention placed on the name recognition of symptoms and pathologies. Particular attention has been given to the grid-based environment, its software architecture and how it may be possible to design a modular application to use GATE functionalities in a grid-based solution.

## Competing interests

The authors declare that they have no competing interests.

## Authors' contributions

MC conceived of the study, and coordinated the design and test of the middleware application and drafted the manuscript. GM participated in the design of bioinformatics application and evaluation. RB participated in the study of knowledge discovery techniques. GT participated in the test of middleware and knowledge discovery study and carried out experimental results for the knowledge discovery application, and helped to draft the manuscript. All authors read and approved the final manuscript.
